# Fluid consumption and taste novelty determines transcription temporal dynamics in the gustatory cortex

**DOI:** 10.1186/s13041-016-0188-4

**Published:** 2016-02-09

**Authors:** Sharon Inberg, Eyal Jacob, Alina Elkobi, Efrat Edry, Akiva Rappaport, T. Ian Simpson, J. Douglas Armstrong, Noam Shomron, Metsada Pasmanik-Chor, Kobi Rosenblum

**Affiliations:** Sagol Department of Neurobiology, Center for Gene Manipulation in the Brain, University of Haifa, Haifa, 3498838 Israel; Center for Gene Manipulation in the Brain, University of Haifa, Haifa, 3498838 Israel; Institute for Adaptive and Neural Computation, School of Informatics, University of Edinburgh, Edinburgh, UK; Biomathematics and Statistics Scotland, James Clerk Maxwell Building, The King’s Buildings, Mayfield Road, Edinburgh, EH9 3JZ UK; Sackler Faculty of Medicine, Bioinformatics Unit, George Wise Faculty of Life Sciences, Tel-Aviv University, Tel-Aviv, Israel

**Keywords:** Arc/Arg3.1, Consolidation, Hydration, Memory, RNA, Temporal dynamics

## Abstract

**Background:**

Novel taste memories, critical for animal survival, are consolidated to form long term memories which are dependent on translation regulation in the gustatory cortex (GC) hours following acquisition. However, the role of transcription regulation in the process is unknown.

**Results:**

Here, we report that transcription in the GC is necessary for taste learning in rats, and that drinking and its consequences, as well as the novel taste experience, affect transcription in the GC during taste memory consolidation. We show differential effects of learning on temporal dynamics in set of genes in the GC, including Arc/Arg3.1, known to regulate the homeostasis of excitatory synapses.

**Conclusions:**

We demonstrate that in taste learning, transcription programs were activated following the physiological responses (i.e., fluid consumption following a water restriction regime, reward, arousal of the animal, etc.) and the specific information about a given taste (i.e., taste novelty). Moreover, the cortical differential prolonged kinetics of mRNA following novel versus familiar taste learning may represent additional novelty related molecular response, where not only the total amount, but also the temporal dynamics of transcription is modulated by sensory experience of novel information.

**Electronic supplementary material:**

The online version of this article (doi:10.1186/s13041-016-0188-4) contains supplementary material, which is available to authorized users.

## Background

Molecular memory consolidation, the post-acquisition phase when a memory is labile and sensitive to interference, is dependent on both transcription and translation in the relevant brain structures in different learning paradigms [1–4]. Long term memories are stored at least partially in the cortex [5, 6]. Thus, revealing the cortical transcription program underlying long-term memory consolidation is a central goal for current neuroscience research.

Taste learning is an insular cortex-dependent behavioral paradigm, which utilizes the innate response of animals toward the nutrient source, characterized by robust, easily controlled and measured behavioral responses [7, 8].

Taste memory consolidation, in a similar way to other learning paradigms, is sensitive to the inhibition of protein synthesis in the gustatory cortex (GC) [9, 10]. Accumulated data have revealed that novel taste learning is associated with different biochemical changes in the GC which resides within the insular cortex and subserves taste memory consolidation in rodents [7, 11]. These changes include increased cholinergic activity [12] and changes in protein phosphorylation state of different proteins and pathways [13–17], for recent review see [18].

Immediate early genes (IEGs) are the first genes to be expressed after external stimulation, and play fundamental roles in synaptic plasticity and cognitive processes including memory consolidation. One IEG that is known to play a major role in excitatory or inhibitory synapse homeostasis of excitatory cells is Activity Regulated Cytoskeleton associated Protein (Arc)/Arg3.1.

Arc/Arg3.1 is used as a reporter of neural activation and synaptic plasticity [19]. Together with its involvement in synaptic and cellular processes, Arc/Arg3.1 is important for consolidation of different forms of synaptic plasticity and long term memory including conditioned taste aversion (CTA) [20].

We hypothesized that transcription in the GC was necessary for novel taste learning and that this would involve a differential transcriptional response. Moreover, the temporal dynamics of gene expression and their involvement in neuronal response to novelty and memory consolidation are poorly understood. In order to test this, we first assessed the effects of the general transcriptional inhibitor, actionmycin D, in the CTA and latent inhibition of CTA learning paradigms and then profiled the transcriptional response to novel taste during the first hours of the consolidation phase- 1 and 3 h post experience, where we expect to find the strongest effect of hydration and learning on the transcription programs. Finally, we measured the dynamic expression of the plasticity related gene- Arc/Arg3.1 following both hydration or novel tastes learning. We have found that the temporal dynamics of transcription is the main discriminant factor between general physiological responses to drinking and novel taste learning.

## Results

### Transcription in the gustatory cortex is necessary for consolidation of both positive and negative forms of taste learning

**Fig. 1:**
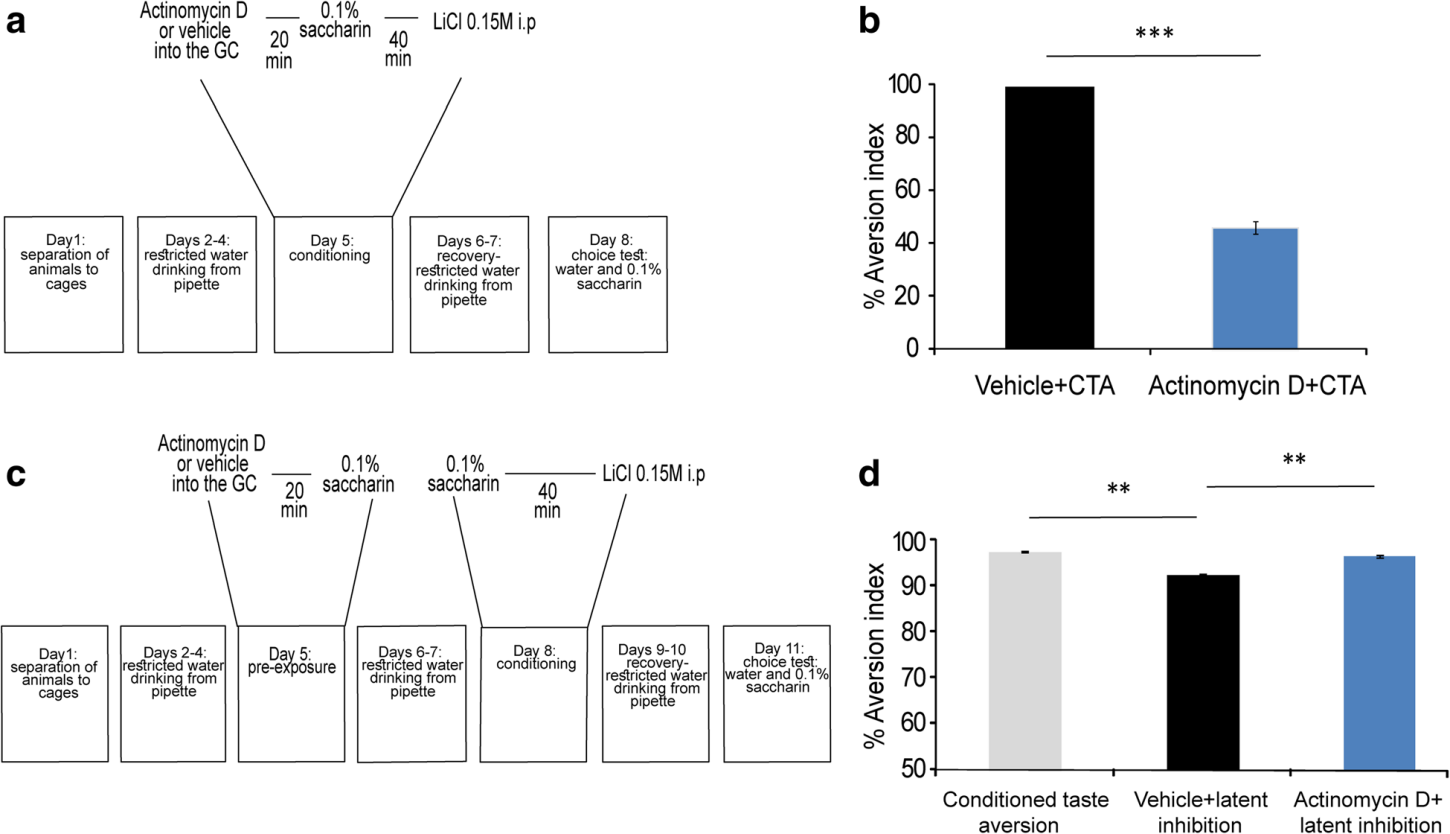
Memory formation for conditioned taste aversion (CTA) and latent inhibition of CTA is dependent on gene transcription in the gustatory cortex. **a** Schematic representation of the behavioral protocol for injection of actinomycin D into the GC, 20 min before drinking of novel taste and i.p. injection with 0.15 M LiCl. **b** Impaired long memory formation, manifested as reduction in aversion index, in rats injected with actinomycin D before CTA learning. Actinomycin D + CTA (*n* = 14); Vehicle + CTA (*n* = 14), *** *p* < 0.0001. **c** Schematic representation of the latent inhibition of CTA procedurefor injection of actinomycin D or vehicle, into the GC during the pre-exposure to novel taste at day 5, . At day 8 all the rats undergo CTA and were tested for memory performance three days later. **d** Impaired latent inhibition of CTA in rats injected with actinomycin D before pre-exposure to novel taste (CTA (*n* = 8); vehicle + latent inhibition (*n* = 14); actinomycin D + latent inhibition (*n* = 14)) ** *p* < 0.001

Taste memory consolidation is protein synthesis dependent [9]. We tested the hypothesis that transcription in the GC is necessary for taste memory consolidation. For this purpose, we stereotaxically injected the widely used, transcription inhibitor actinomycin D or vehicle as a control [21] (1 μl, 20 ng/μl) into the GC, 20 min before CTA (Fig. 1a).

The results show that transcription inhibition in the GC impairs CTA learning, manifested as reduction in aversion indices presented as percentage (actinomycin D: 46.00 ± 2.34 %; Vehicle: 98.82 ± 0.23 %; Mann–Whitney, z = −4.50, *p* = 5 × 10^−8^, *n* = 14 for both vehicle and actinomycin D groups, Fig. 1b), indicating a strong long term memory impairment in actinomycin D treated animals. At the very same time, the transcription inhibition did not affect total fluid consumption at the conditioning day (Additional file 1: Figure S1A). These results suggest that transcription is essential for associative taste learning in the GC.

Moreover, injection of actinomycin D into the GC, 20 min before pre-exposure to 0.1 % saccharin (Fig. 1c), impaired a positive form of taste learning. i.e., the latent inhibition of CTA (CTA: 97.28 ± 0.28 %; latent inhibition + vehicle: 92.37 ± 0.23 %; latent inhibition + actinomycin D: 96.49 ± 0.13 %; ANOVA: *p* < 0.00013, F2 = 13.63, Tukey HSD post-hoc: CTA to latent inhibition + vehicle, *p* < 0.0001; latent inhibition + vehicle to latent inhibition + actinomycin D, *p* < 0.001. *n* = 14 for latent inhibition groups, *n* = 8 for CTA group, Fig. 1d). Actinomycin D is toxic, in some circumstances [22]. However, it is a widely used transcription inhibitor in memory consolidation field [2].

### Consumption of fluids and not information about taste familiarity dominantly affects transcription in the gustatory cortex

**Fig. 2:**
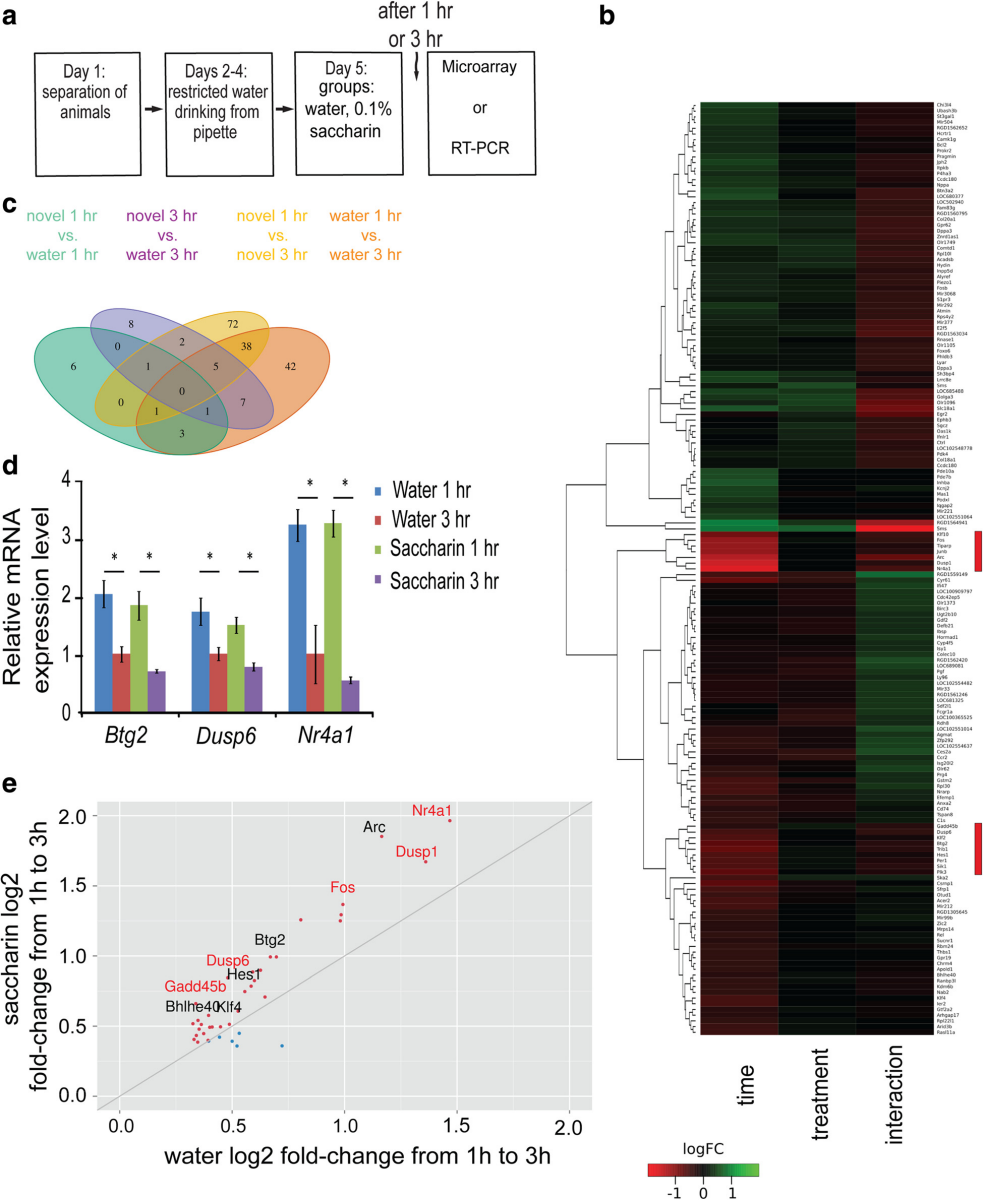
Time from fluid consumption dominantly affects transcription in the gustatory cortex. **a** Schematic representation of the behavioral paradigm of novel taste learning, in which the animal received familiar taste (water) or novel taste (0.1 % saccharin) and was scarified 1 h or 3 h later. **b** Heatmap for gene expression. Individual microarray chips cluster by time and not treatment (novel saccharin and familiar water). Heatmap scale: Red- higher expression of transcripts at 1 h compared to 3 h (“Time”. For the same taste comparison) and higher for water compared to saccharin (“Treatment”. For the same time point comparison). Green- higher expression of transcripts at 3 h compared to 1 h (“Time”. For the same taste comparison) and higher for saccharin compared to water (“Treatment”. For the same time point comparison). The red rectangles at the right side mark the transcripts with the highest fold change. **c** Venn diagram with 4 possible comparisons within and between time points and treatments. More genes are regulated in the 1 to 3 h temporal dynamics than at each pecific time point. *n* = 4 in each group. Cut-off: *p* < 0.05, 0.8 ≥ absolute fold change ≥1.25. **d** Btg2, Dusp6 and Nr4a1 qRT-PCR validation. The Btg2, Nr4a1, and Dusp6 genes were identified as strongly modulated by drinking as described in Fig. 2b, c. These genes were among the 44 genes in the common list of the 1 to 3 h temporal dynamics, following drinking of novel 0.1 % saccharin and water in the GC (*n* = 4 for all groups; * *p* < 0.05). **e** More genes from the common list are differentially modulated in the temporal dynamics of the novel taste group, compared to water group. 32 out of 38 common genes list (for *p* < 0.0001) have higher fold change values in the novel taste group. Genes with higher fold change in saccharin- red dots; higher fold change in water- blue dots. Genes from MAPK pathway coloured black

In order to identify the modulated genes correlated with taste learning, we used microarray technology (16 Affymetrix GeneChip, RaGene 1.1 ST v1) to profile gene expression in the GC following novel taste learning. For this purpose, rats were exposed to either novel taste (0.1 % saccharin) or a familiar taste (water) and were sacrificed either 1 h or 3 h following the end of drinking session (Fig. 2a, *n* = 4) (no significant difference in the amount of consumed taste was observed between water and 0.1 % saccharin, at any time point, Additional file 1: Figure S1B). For each animal, GC-derived cDNA samples were prepared, in-vitro transcribed to produce biotinylated cRNA and hybridised to individual microarray slides, in accordance with manufacturer’s instructions (Affymetrix, Santa Clara, CA, USA).

Hierarchical clustering analysis, represented by heatmap for gene expression following novel taste learning revealed that the time following drinking (1 h and 3 h), but not the treatment (the identity of the consumed taste, i.e., novel or familiar taste (Fig. 2b) is the main factor that differentiate novel taste from familiar one.

Next, quantification of the number of differentially expressed genes was made and presented by the Venn diagram at Fig. 2c (cut-off: *p* < =0.05,0.8 > =fold change > =1.25). Very few genes were differentially modulated, in the comparison between novel taste and familiar taste group, at both 1 h and 3 h time points. In total, expression levels of 12 and 24 genes at the 1 h and 3 h time point, accordingly, were differentially modulated. However, when we analyzed the temporal expression dynamics by comparing differentially expressed genes at 1 h and 3 h time points for each treatment group, we found unique 75 genes in the novel condition and 53 in the familiar group. In addition, 44 genes were common to the dynamics of both treatment groups and probably related to the effect of drinking itself. All in all, 4 to 5 times more genes were modulated in the temporal dynamic comparison than at each specific time point for familiar and novel taste groups (Additional file 2: Table S1 and Additional file 3: Table S2).

Validation of the 1 to 3 h temporal dynamics of candidate genes from the common list (44 genes, Fig. 2c) with relative high fold change and low *p*-value were done using qRT-PCR. Specifically, Dusp6, Nr4a1 and Btg2 were down-regulated in 3 h compared to 1 h, in both, novel and familiar tastes (Dusp6: water 1 h: 1.73 ± 0.24, water 3 h 1.00 ± 0.12Mann Whitney test, one tail test: *p* < 0.05, z = −2.3; saccharin 1 h: 1.79 ± 0.14 0.1 % saccharin 3 h: 0.87 ± 0.07, Mann Whitney test, one tail test: z = −2.3: *p* < 0.05; Nr4a1: water 1 h: 3.21 ± 0.27, water 3 h 1.00 ± 0.50: Mann Whitney test, one tail test: water, z = −2.3: *p* < 0.05; saccharin 1 h: 3.24 ± 0.23, saccharin 3 h: 0.55 ± 0.05, Mann Whitney test, one tail test: z = −2.3, *p* < 0.05; Btg2: water 1 h: 2.03 ± 0.24, water 3 h 1.00 ± 0.13 ± 0.13, Mann Whitney test, one tail test: z = −2.3, *p* < 0.05; saccharin 1 h: 1.83 ± 0.25183 ± 0.25, saccharin 3 h: 0.70 ± 0.03, Mann Whitney test, one tail test: z = −2.3, *p* < 0.05 Fig. 2d).

A closer look at the magnitude of differential expression for the 44 (cut-off: *p* < =0.05, 0.8 > =fold change > =1.25) shared dynamic genes for both novel and familiar taste, reveals a strong expression bias towards novel taste. Out of the 44 genes, 38 are up-regulated and 32 of these show greater induction between 1 h and 3 h with a novel taste compared to a familiar one (only 6 genes, Fig. 2e). This suggests that in addition to the drinking-dependent effects, there are also distinct temporal transcriptional signals that differ by the identity and the familiarity of the consumed taste. This important distinction between hydration and novel taste- the temporal dynamics of transcription levels will be the focus of the following parts.

### Novel taste learning differentially affects temporal dynamics of the transcriptome in the GC

**Fig. 3:**
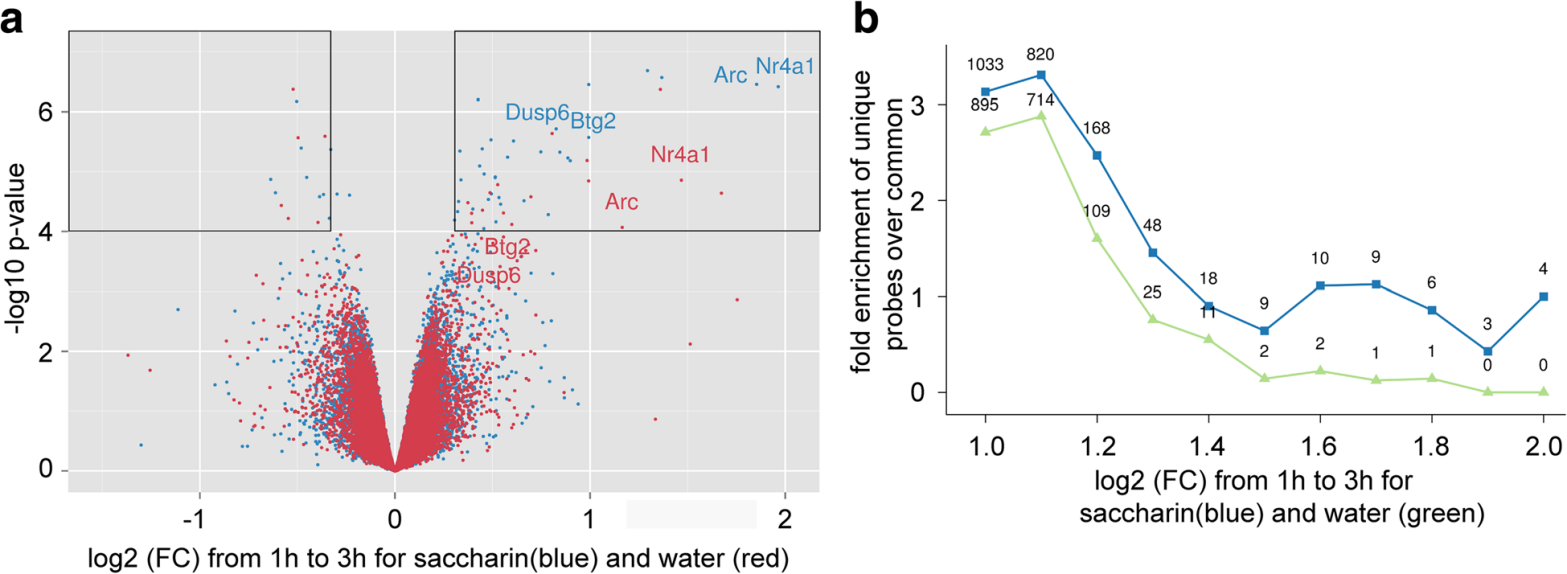
Novel taste affects dynamics of transcription differentially from familiar taste. **a** Volcano plot for gene expression’s temporal dynamics- 1 h to 3 h. Novel taste learning with 0.1 % saccharin induced more differentially expressed genes than familiar water. In addition, more genes in the saccharin group are higher in 1 h compared to 3 h. Arc/Arg3.1, Btg2, Dusp6 and Nr4a1 in both water and novel 0.1 % saccharin groups marked. The black boxes represent the cut-off limits for the p-value and fold change. **b** Comparison between the amounts of differentially expressed genes for each fold change normalized to the number of genes from the common list. Novel taste learning induced more differentially modulated genes in the 1 h to 3 h temporal dynamics compared to water for *p* < 0.05 and various fold change values described in X axis. The values on the graph represent the number of differentially expressed genes for each fold change

We next compared the distribution of the differential transcriptome dynamics in each treatment. In agreement with Fig. 2e, the volcano plot (Fig. 3a) revealed unequal distribution of total screened genes extracted from the GC following experience of familiar or novel taste. Specifically, more genes were down-regulated between the 1 and 3 h time points (i.e., their expression is lower in 3 versus 1 h, positive values in x-axis) compared to up-regulated genes (i.e., their expression is higher in 3 versus 1 h, negative values in x-axes). In order to quantify the number of genes differentially expressed between novel and familiar tastes, cut-off criteria were defined as *p*-value < 0.0001 (−log10(p) > 4) and fold change higher than 1.25 (−0.325 > log2(FC) > 0.325), marked with black rectangles in Fig. 3a. In total, 46 unique genes were found in the novel taste group, versus 19 unique genes in the familiar taste group. Moreover, in the novel taste group, 35 genes were down-regulated between the 1 h and 3 h time points, whereas only 11 genes were up-regulated in 3 h. In the familiar taste group 14 genes were down-regulated between the 1 h and 3 h time points, whereas 5 genes were up. These results indicate that more genes are changing in the novel taste group in parallel to the asymmetrical direction of the change, with significantly more genes higher at 1 h compared to 3 h in the novel taste group.

The 1 h to 3 h temporal dynamics of gene expression in novel and familiar taste differ in the number of genes involved, as can be seen from the volcano plot (Fig. 3a). In order to study the effects of fold-change cut-off on the number of differentially expressed genes between novel and familiar taste groups we varied the cut-off and calculated the enrichment of unique genes normalized to the number of common genes. We found that at all cut-off values more genes were differentially expressed in the novel taste group compared to the familiar taste group, an effect strengthened with the increase in fold change (Fig. 3b). These results indicate that novel taste learning induced greater changes in the transcriptional temporal dynamics of the GC compared to familiar taste. Overall, the temporal dynamics of gene expression specified as a major component that differentiate between hydration and novelty processing (Additional file 2: Table S1 and Additional file 3: Table S2).

### Novel taste learning decreases the expression of Arc/Arg3.1 in the gustatory cortex for many hours

Following the transcriptome analysis, we aimed at looking for the detailed expression of Arc/Arg3.1 since it is involved in memory consolidation [23].

**Fig. 4:**
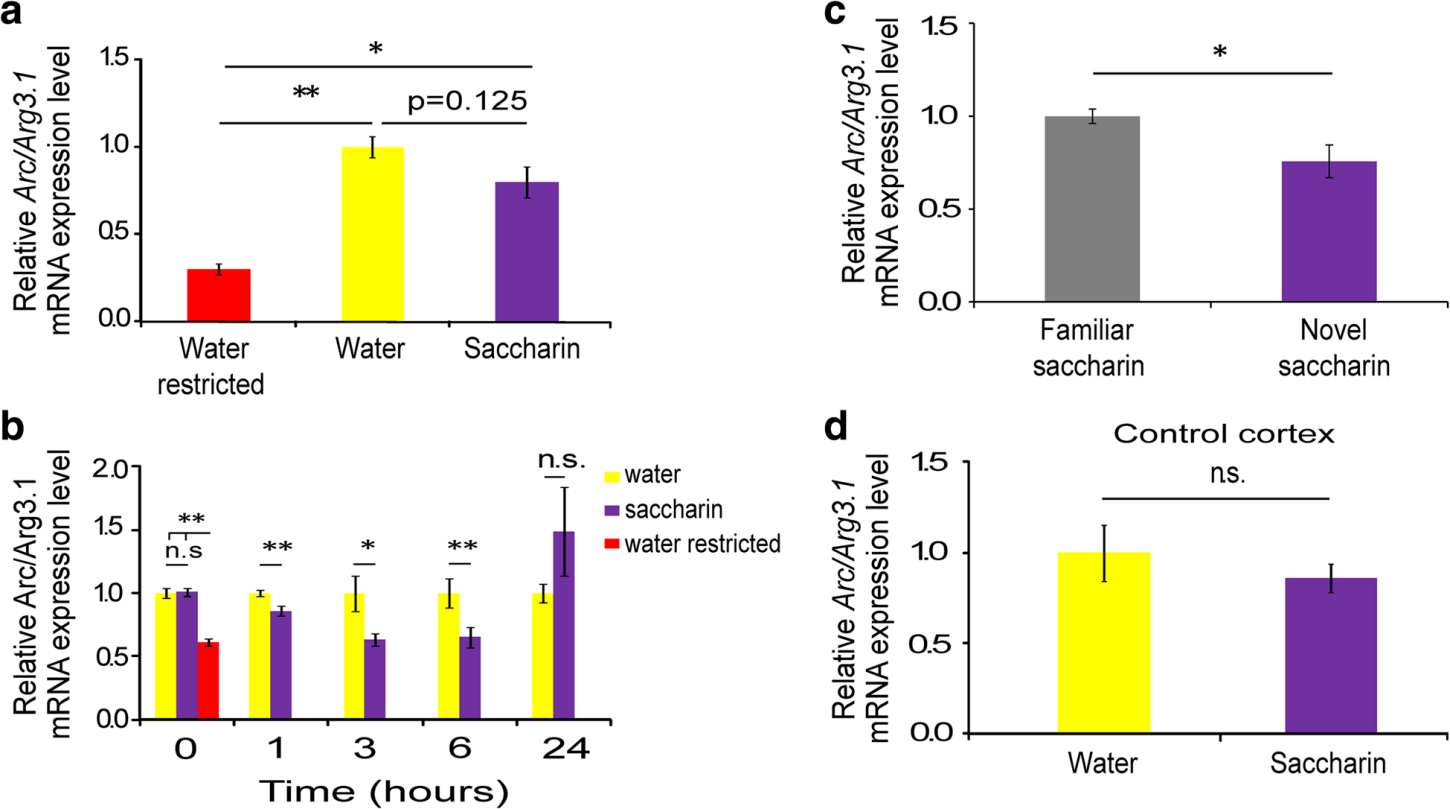
Novel taste decreases the expression of Arc/Arg3.1 in the gustatory cortex for few hours. **a** 1 h following drinking Arc/Arg3.1 mRNA levels were elevated in both the water (*n* = 5) and 0.1 % saccharin groups (*n* = 6) compared to the water restricted (*n* = 5) group *p < 0.05, **p < 0.01. **b** Novel taste learning induced reduction in Arc/Arg3.1 mRNA expression hours following learning. Immediately after the end of drinking session- 0 h (water, *n* = 11; saccharin, *n* = 11; water restricted, *n* = 11), 1 h (water, *n* = 20; saccharin, *n* = 20), 3 h (water, *n* = 8; saccharin, *n* = 9), 6 h (water, *n* = 14; saccharin, *n* = 14), 24 h (saccharin, *n* = 11; water, *n* = 12) * *p* < 0.05, ** *p* < 0.01. **c** Novel taste learning reduced the amounts of Arc/Arg3.1 mRNA expression, compared to 25 days of familiar saccharin group (1 h: familiar saccharin, *n* = 5; novel saccharin, *n* = 5) * *p* < 0.05. **d** Arc/Arg3.1 expression does not change in the non-relevant occipital cortex 6 h following novel taste learning (saccharin, *n* = 6; water, *n* = 6, *p* = 0.82)

The transcriptome dynamics suggested that drinking itself has the major effect on gene expression, including Arc/Arg3.1, which has one of the highest fold change in the volcano plot (Fig. 3a) and is involved in novel taste learning [24]. To test directly the effect of hydration, we added a water restricted group, which underwent 3 days of restricted drinking schedule (meaning 24 h of water restriction which reflects the basal level of gene expression before consumption familiar or novel taste). Expression levels of the Arc/Arg3.1 were higher after 1 h in the two drinking groups compared to the water restricted group (Arc/Arg3.1 (Fig. 4a): water: 1.00 ± 0.06; saccharin:0.80 ± 0.09; water restricted: 0.30 ± 0.03, main effect of the group, ANOVA, F_(2,16)_ = 25.9, *p < 0.0001,* post-hoc Tukey HSD-water restricted vs. water *p = 0.000027,* post-hoc Tukey HSD- water restricted vs. saccharin *p < 0.001,* post-hoc Tukey HSD- *water vs. saccharin p = 0.125).*

Broader examination, with more independent experiments, of Arc/Arg3.1 mRNA expression levels at different time points following novel taste learning revealed the temporal dynamics of Arc/Arg3.1 expression. Immediately after drinking (defined as time 0 h) there is no significant difference between novel and familiar taste groups, but significant increase in both groups compared to the water restricted group (Arc/Arg3.1 (Fig. 4b: water: 1.00 ± 0.04; saccharin:1.01 ± 0.03; water restricted: 0.610 ± 0.03, main effect of the group, ANOVA, F_(2,16)_ = 4.32, *p < 0.05,* post-hoc Tukey HSD -water restricted vs. water *p = 0.044,* post-hoc Tukey HSD -water restricted vs. saccharin *p < 0.05,* Tukey HSD- water vs. saccharin *p = 1.00)*.

The differential effect of novelty on Arc/Arg3.1 expression, in the GC, emerges 1 h following learning and last for at least 5 h (Arc/Arg3.1: 1 h

water: 1.00 ± 0.03; saccharin:0.86 ± 0.04, *t*-test, t30 = 3.3, *p* < 0.01; 3 h: water: 1.00 ± 0.13; saccharin: 0.66 ± 0.05, *t*-test, *t*_15_ = 2.5, *p* < 0.05; 6 h: water: 1.00 ± 0.11; saccharin:0.65 ± 0.08, Mann Whitney: z = −2.5, *p* < 0.05). 24 h following learning, no significant difference was observed between the two groups (water: 1.00 ± 0.07; saccharin: 1.49 ± 0.35, Mann Whitney: z = −0.74, *p* = 0.487, Fig. 4b). We thus conclude that there are two phases of Arc/Arg3.1 response- fast, taste independent upregulation followed by taste dependent temporal dynamics of gene expression.

In order to show that the response of Arc/Arg3.1 mRNA is specific to the novelty of the taste and not to its chemical identity, another group of rats undergo 25 days of familiarization to saccharin and were compared to novel saccharin group. We found a significant decrease in the amounts Arc/Arg3.1 mRNA levels in the novel taste group, compared to familiar saccharin (Ffamiliar saccharin: 1.00 ± 0.039; Novel saccharin: 0.76 ± 0.089, *t*-test, t8 = 2.51, *p* < 0.05, Fig. 4c). Following that, we conclude that Arc/Arg3.1 mRNA reduction is novelty specific.

In order to test if the modulation in Arc/Arg3.1 expression is specific to the GC, we analyzed its expression 6 h after novel taste learning in the occipital cortex (OC), and found no changes between novel and familiar taste (Arc/Arg3.1: water: 1.00 ± 0.15; saccharin:0.86 ± 0.08, Mann Whitney: z = −0.32, *p* = 0.82; Fig. 4d), indicating GC-specific differential effect of novel taste learning.

## Discussion

Since long term memory formation requires RNA synthesis [2, 25], we aimed to test the involvement of RNA transcription in cortical dependent taste learning. Our results showed that local application of RNA synthesis inhibitor, actinomycin D, into the gustatory cortex strongly impairs memory formation for CTA and latent inhibition of CTA, similarly to the effect of protein synthesis inhibition [9]. This suggests that intact RNA synthesis in the GC is required for the consolidation of GC-dependent taste learning paradigms.

To date, gene expression at the transcriptional level in the GC following novel taste learning has not been reported in the literature. Therefore, we attempted to recognize specific genes modulated following novel taste learning, by performing microarray screening on GC samples of rats exposed to novel or familiar taste, and sacrificed 1 or 3 h later. While 0.1 % saccharin was used in the current study as a novel taste, previous work have shown that the molecular machinery in the GC is responding to other novel tastes (e.g. sodium chloride) in a very similar way [24].

In contrast to our hypothesis that the clustering of gene expression would be according to registered information about the taste, i.e., novel or familiar at each time point, we found clustering by time, 1 and 3 h following the drinking session. This strong clustering by time following hydration indicates that the effect of drinking and its consequences (changes in blood osmolarity, arousal response, reward-related processes, etc.) on transcription is stronger than that of learning a novel taste, when the two groups are compared by time point.

Drinking after water deprivation has a strong effect on the physiology of the brain, for instance, when water content increased in the blood (hyposmolality) it equilibrates within minutes across the blood–brain barrier and brain cell membranes. The result is swelling of neurons and glia cells [26]. Hydration decreased plasma osmolarity and increased sodium concentration in the plasma of Sprague–Dawley rats [27]. In humans, changes in the hydration state affect morphological features of white matter, gray matter, and cerebrospinal fluid levels [28].

The transcription program we identify in the cortex of water restricted rats following drinking reflects the massive differential activity in the brain and may explain susceptibility to seizure following hydration and less sensitivity to seizure following dehydration [25]. In addition to the clinical aspects of the results, it is possible that hydration itself is a major factor in some of the correlative results obtained in water restricted animals which use drinking as positive reinforcement [29, 30].

In spite of the relatively small number of differentially expressed genes at each time point, novel taste learning can be distinguished from hydration effects with a familiar taste by unique temporal dynamics patterns of general gene expression and specifically the IEG Arc/Arg3.1. These patterns consist of the expression of more genes with higher fold change following novel taste consumption compared to water, and more genes whose temporal dynamics were differentially modulated following novel taste consumption. Following taste learning, the hydration influence on gene transcription profile (as indicated by strong clustering at 1 and 3 h) preceded the learning-induced transcription profile (as indicated by 1 h to 3 h gene expression temporal dynamics). By injecting actinomycin D into the gustatory cortex prior to taste learning, we probably interrupted with both the physiological transcriptional response to hydration and to novelty.

Interestingly, Cavallaro et al. [31] found using the Morris water maze paradigm, different patterns of gene expression across time, differentiating between physical activity (swimming) and memory consolidation. Both in the study by Cavallaro et al. (2002) and in the current study, there are at least two gene expression programs: learning-specific (differential temporal dynamics) and learning non-specific (clustering by hydration effects at each time point).

Since the specificity of the cellular response to external stimuli is dependent on precise temporal control and relative spatial distribution of activated signals [32], we suggest that by different durations or dynamics of transcription activation, the molecular machinery responds differentially to novel and familiar taste on the background of the initial strong and non-specific effects of hydration.

Following the results from the microarray screen, we examined the expression of IEG Arc/Arg3.1 in the GC at different time point during the 24 h following novel taste learning.

The results show that transcription of Arc/Arg3.1 is dual-phased, where first (time 0) it undergo hydration-dependent and taste-independent transcription regulation, which is followed by a second, taste learning-dependent phase of regulation with long lasting decrease in mRNA levels of Arc/Arg3.1 transcript following novel taste learning.

Arc/Arg3.1 expression and its temporal dynamics reflects general gene transcription, with an initial strong elevation (2.5 to 3 fold) following hydration. This first physiological response was followed by a smaller, differential effect of novelty dependent in the temporal dimension for up to at least 6 h, influencing the new transcription content generated following hydration.

Taken together, these two sequential phases may represent two continuous phases of physiological and sensory input processing. We showed that the drinking associated phase represented by immediate increase in transcription, followed by a novelty specific altered processing of the new transcriptional content, demonstrated by differential temporal dynamics compared to general hydration related transcriptional response.

The transcriptional response to hydration for both the IEG and the general transcription program components can be part of impulse-like changes in mRNA levels following external input such changes in cellular osmolarity, reward-related processes and arousal. These impulse responses are molecular programs, involved in encoding and decoding of information into specific cellular responses following different external stimulations such as heat shock, oxidative stress, response to pathogens and osmotic pressure [33].

Arc/Arg3.1 plays important roles in synaptic plasticity. In the synapse, Arc/Arg3.1 is involved in α-amino-3-hydroxy-5-methyl-4-isoxazolepropionic acid receptor (AMPA-R) endocytosis [34]. In the nucleus, Arc/Arg3.1 is involved in GluA1 transcription inhibition, leading to downscaling of synaptic strength [35]. Arc/Arg3.1 is strongly up-regulated in many brain regions following various behavioral paradigms [36–38]. However, Arc/Arg3.1 down-regulation following learning has been observed only in the hippocampal dentate gyrus in rats repeatedly exposed to an empty arena in an object recognition memory paradigm [39]. The long lasting decrease of Arc/Arg3.1 mRNA in the group exposed to novel taste (starting at 1 h) may be ascribed to an increased degradation rate of Arc/Arg3.1 mRNA [40]. As above-mentioned, this prolonged decrease is preceded by fluid consumption-induced strong and rapid increase in Arc/Arg3.1 mRNA levels. We suggest that the novel taste-induced prolonged reduction in Arc/Arg3.1 mRNA levels compared to familiar water, possibly through RNA degradation machinery, is a network/cellular adjustment to elevated levels of Arc/Arg3.1, in order to reach the optimal range of Arc/Arg3.1 mRNA expression for normal synaptic plasticity functions [30]. Since in some occasions Arc/Arg3.1 expression is negatively correlated with learning, it was suggested that higher levels of Arc/Arg3.1 may slow down acquisition of new information [30, 41]. Furthermore, these authors suggested that since Arc/Arg3.1 is associated with the cytoskeleton and the post synaptic density, high Arc/Arg3.1 mRNA expression levels result in synaptic architecture over stabilization.

## Conclusions

Temporal dynamics of gene transcription in the GC is part of gene expression programs initiated following consumption of novel taste. The temporal dynamics of gene expression is one additional form of molecular response to novel sensory experience, in the relevant cortical area. The data for Arc/Arg3.1 and other transcripts described in the microarray screen, suggest that on the background of altered transcriptional content following hydration, the temporal dynamics of transcription reflects more by experiencing novel information, in contrast to the total amounts of transcription at each specific time point that represent general response to hydration.

## Methods

### Animals

Wistar Hola male rats, 10–16 weeks old, were used in all experimental procedures. The rats were caged individually at the beginning of the behavioral session and placed in a 12 h light and 12 h dark cycle at a constant temperature of 22 °C, with standard rat chow and tap water available ad libitum, except during the experiment, when animals were water deprived 24 h prior to daily ration of fluids and between daily rations delivered through pipettes throughout the experiment. All experiments were performed in the light phase.

Animals were handled according to approved protocols and animal welfare regulations of the Institutional Animal Care and Committee of the University of Haifa. Protocols were also in accordance with the guidelines laid down by the Israeli National Institutes of Health.

### Behavior

For all behavioral paradigms, animals were water restricted and housed separately in a new cage 24 h before beginning of pipette drinking schedule. Food was available ad libitum throughout the experiment.

### Incidental taste learning

Following separation from grouped home cages, rats were subjected to water restriction regime and were trained to drink their daily 20 ml of water from two 10 ml plastic pipettes for the duration of 20 min for 3 consecutive days. Following training, animals were divided into two groups: water as a familiar taste and 0.1 % saccharin as a novel taste.

Consumption of at least 10 ml is required in order to achieve learning [10]. Animals which did not reach the minimum amount in five additional minutes were excluded from the experiment. Animals were decapitated for biochemical analysis at several time points following the end of the drinking session: Immediately following the end of 20 min drinking (time 0), 1, 3, 6, and 24 h. In addition, we added a water restricted group with no access to fluids for 24 h until decapitation.

### Long lasting familiarization with saccharin

The familiarizaton assay was used as described previously [24]. Saccharin (0.1 %) was available ad libitum from a bottle for 21day in a grouped cage (6 rats). Animals were then separated to individual cages and allowed access to 0.1 % saccharin from pipette restricted to 20 ml, 20 min per day for 3 days. On the 25th day the animals drank 0.1 % saccharin or novel 0.1 % saccharin for the second group, from two pippetes and were killed 1 h after the end of the drinking session for further analysis.

### Conditioned taste aversion and actinomycin D injections

Rats were cannulated in the GC as previously described [24] and allowed one week for recovery. Following recovery, the cannulated rats were subjected to the water restriction regime and were trained to drink their daily 20 ml of water from two 10 ml pipettes for a duration of 20 min for 3 consecutive days.

On the 5th day, the conditioning day, the animals were injected bilaterally with 1 μl of actinomycin D (20 ng/μl) dissolved in 0.2 % Dimethyl sulfoxide (DMSO) or vehicle (0.2 % DMSO) into the GC. Twenty minutes later they were allowed to drink the 0.1 % saccharin solution from pipettes for 20 min, and 40 min following the cessation of the drinking period they were injected with 0.15 M LiCl i.p. In order to recover, the animals drank water in the subsequent two days and on the third day after CTA they underwent a multiple-choice test in which they were offered two pipettes, each containing 5 ml of saccharin and two pipettes, each containing 5 ml of water. In order to measure the aversive memory, aversion index was calculated and described as percentage as follows:

$$ \left[\mathrm{ml}\ \mathrm{water}/\left(\mathrm{ml}\ \mathrm{water}+\mathrm{ml}\ \mathrm{saccharin}\right)\right]*100 $$

### Latent inhibition and actinomycin D injections

Rats were cannulated in the GC as previously described [24] and allowed one week for recovery. Following recovery, the cannulated rats were subjected to the water restriction regime and were trained to drink their daily 20 ml of water from two 10 ml pipettes for a duration of 20 min for 3 consecutive days.

On the 5th day, the saccharin pre-exposure day, the animals were injected into the GC bilaterally with 1 μl of actinomycin D dissolved in 0.2 % Dimethyl sulfoxide (DMSO) or vehicle as a control (0.2 % DMSO). Twenty minutes later they were allowed to drink the 0.1 % saccharin solution from pipettes for 20 min. After 2 days of water drinking the animals were subjected to CTA paradigm (40 min interval between the drinking and the i.p injection of 0.15 M LiCl, as described in the previous session). In order to recover, the animals drank water in the subsequent two days and on the third day after CTA they underwent a multiple-choice test in which they were offered two pipettes, each containing 5 ml of saccharin and two pipettes, each containing 5 ml of water. In order to measure the aversive memory, aversion index was calculated and described as percentage as follows [ml water/(ml water + ml saccharin)]*100.

### Microinjection

The stylus was removed from the guide cannula and a 28-gauge injection cannula, extending from the tip of the guide cannula, was carefully placed. The injection cannula was connected via PE20 tubing to a Hamilton micro-syringe, driven by a microinjection pump (CMA/100; Carnegie Medicin, Stockholm, Sweden), 1 μl of Actinomycin D (20 ng/μl) or vehicle was injected. Following injection, the injection cannula was left for an additional 30 s before withdrawal, to minimize liquid retraction.

### RNA extraction

Total RNA was extracted from insular cortex or occipital cortex specimens using RNeasy lipid tissue kit (QIAGEN) (Venlo, Netherlands) according to the manufacturer’s instructions. Total RNA was reverse-transcribed using high capacity cDNA reverse transcription kit (Applied Biosystems, *Carlsbad, CA*, USA).

RNA concentration was quantified using a NanoDrop 2000 spectrophotometer (Thermo Scientific).

### Microarray experiment and data analysis

Affymetrix GeneChip RaGene 1.1 ST v1 arrays were used for gene expression analysis, according to the instruction manual (Affymetrix, Santa Clara, CA, USA).

Two time points- 1 h, 3 h and two treatments- familiar water and novel 0.1 % saccharin were used, 4 chips each, 16 chips in total. The Affymetrix raw CEL files were processed and quality controlled using the R/Bioconductor package ‘affy’[42]. The probe level data from all chips were then quantile cross-normalised and their expression values summarised at the probe set level in log2 scale using the robust multi-chip average (RMA) method [43]. Differentially expressed probe sets were identified using two modelling approaches using the R/Bioconductor package ‘limma’ that implements an Emperical Bayes moderated t-statistic approach to better estimate the error in comparisons. The first analysis compared all relevant contrasts isolated by time and treatment (n1f1, n3f3, n1n3 and w1w3) and the second performed a classical 2-way ANOVA analysis with the conditioning variables of “time”, “treatment” and the interaction term between the two. Probe sets mapping to only one gene were determined from oligo-level alignment of probe sequences to the latest draft of the rat genome (RGDv3.4 assembly). A range of different cut-off criteria were used to capture the expression signatures. 1. Highly differentially expressed genes (*p* < =0.05, 0.8 > =fc > =1.25). 2. all differentially expressed genes (*p* < =0.05) and 3. all genes (retaining differential expression statistics for later use). As part of the quality control process chip-wise hierarchical clustering was performed to determine how chips partitioned by condition using the R/Bioconductor package‘pvclust’ that uses a multi-scale bootstrap re-sampling approach to calculate cluster stability [44]. Comparisons between lists of differentially expressed genes were quantified and visualised using the CRAN package ‘VennDiagram’. Data have been deposited in NCBI GEO, accession number GSE74546.

### Real time PCR

Quantitative real-time PCR analysis was performed using the PCR System STEP-ONE plus (PE Applied Biosystems, Foster City, CA, USA). qRT-PCR reactions were carried out in a total volume of 10 μL on 10 ng of cDNA using the following Taqman® assays (Applied Biosystems): Activity-Regulated Cytoskeleton-associated protein (Arc/Arg3.1, Rn00571208_g1), BTG family, member 2 (Btg2,Rn00568504_m1), glyceraldehyde-3-phosphate dehydrogenase (Gapdh, Rn01775763_g1), Nuclear receptor subfamily 4, group A, member 1 (Nr4a1, Rn01533237_m1), Dual specificity phosphatase 6 (Dusp6, Rn00518185_m1). Relative mRNA levels were calculated using the comparative C_t_ method, using Gapdh as a normalizing gene.

### Statistical analysis

Results are expressed as means ± SEM.

Normal distribution of the data was the main criteria for using parametric tests (*t*-Test, ANOVA), otherwise an equivalent a-parametric tests were used.

For multiple comparisons of RT-PCR ratios: ANOVA tests with Tukey HSD post hoc correction was used for comparing familiar taste, novel taste and water restricted groups.

For two independent groups comparison of RT-PCR ratios: Student’s *t*-test was used to compare familiar taste group to novel taste at each specific time point. In cases where the results were not normally distributed, a-parametric Mann Whitney tests were used for comparing the two groups.

The time course experiments for Arc/Arg3.1 were independent at each time point, with novel and familiar taste as a control group, hence their statistical analysis was in pairs for each specific time point, that represents an independent experiment.

## Additional files

Additional file 1: Figure S1A. Drinking volumes (ml) of novel 0.1 % saccharin 20 min following actinomycin D or vehicle injection into the GC are not significantly different (*n* = 14 for both groups, *p*= > 0.05). Boxplots show the median of the distribution (dark thickened middle line), the 75th percentile (upper limit of box), and 25th percentile (lower limit of box). The whiskers indicate the minimum and the maximum values of each experimental group (vehicle and actinomycin D injected rats). **Figure S1B**. Drinking volumes (ml) of water and novel 0.1 % saccharin groups at various time points used for molecular correlations are not significantly different (1 h: saccharin (*n* = 23), water (*n* = 22), *p* > 0.05; 3 h: saccharin (*n* = 9), water (*n* = 8*, p* > 0.05; 6 h: saccharin (*n* = 13), water (*n* = 13), *p* > 0.05; 24 h: saccharin (*n* = 9), water (*n* = 9), *p* > 0.05. Boxplots show the median of the distribution (dark thickened middle line), the 75th percentile (upper limit of box), and 25th percentile (lower limit of box). The whiskers indicate the minimum and the maximum values of each experimental group at each time point (1, 3, 6, 24 h). (TIF 844 kb) Additional file 2:
**Expression of mRNAs in the GC.** Novel saccharin compared to water group, between and within group comparison at 1 and 3 hrs. (XLSX 39 kb) Additional file 3:
**ANOVA differential expression.** The influence of time (1,3 hrs), treatment (novel saccharin, water) and the interaction between the two on mRNA expression in the GC. (XLSX 31 kb)

## Abbreviations

GC: Gustatory cortex
IEG: Immediate early genes
Arc/Arg3.1: Activity regulated cytoskeleton associated protein
CTA: Conditioned taste aversion
