# Supplementary figures and images for: Fluid consumption and taste novelty determines transcription temporal dynamics in the gustatory cortex
Source: Mol Brain. 2016 Feb 9;9:13. doi: 10.1186/s13041-016-0188-4 (PMC4746785; doi:10.1186/s13041-016-0188-4)

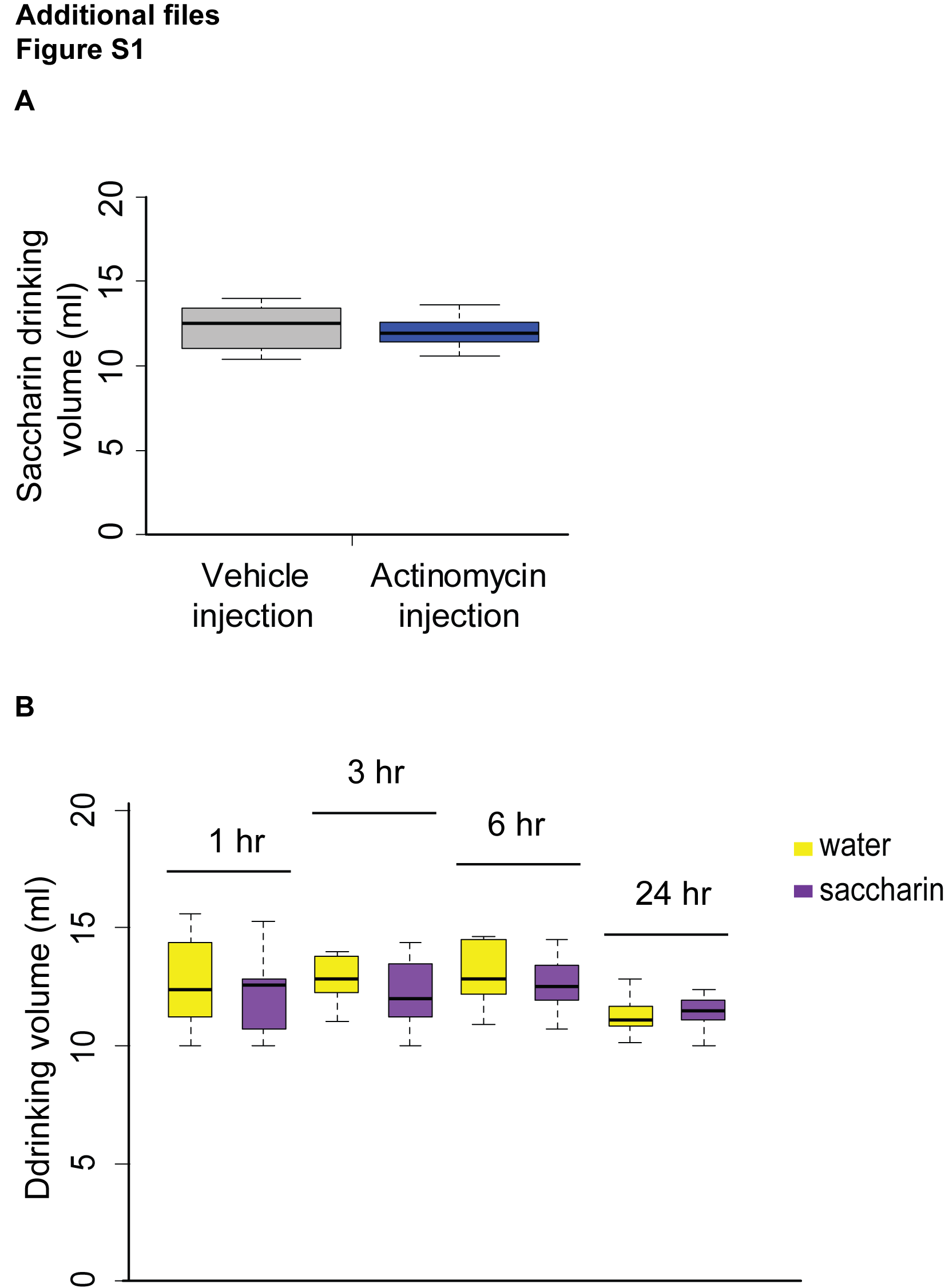

Supplement: Additional file 1: Figure S1A. — Drinking volumes (ml) of novel 0.1 % saccharin 20 min following actinomycin D or vehicle injection into the GC are not significantly different (n = 14 for both groups, p= > 0.05). Boxplots show the median of the distribution (dark thickened middle line), the 75th percentile (upper limit of box), and 25th percentile (lower limit of box). The whiskers indicate the minimum and the maximum values of each experimental group (vehicle and actinomycin D injected rats). Figure S1B. Drinking volumes (ml) of water and novel 0.1 % saccharin groups at various time points used for molecular correlations are not significantly different (1 h: saccharin (n = 23), water (n = 22), p > 0.05; 3 h: saccharin (n = 9), water (n = 8, p > 0.05; 6 h: saccharin (n = 13), water (n = 13), p > 0.05; 24 h: saccharin (n = 9), water (n = 9), p > 0.05. Boxplots show the median of the distribution (dark thickened middle line), the 75th percentile (upper limit of box), and 25th percentile (lower limit of box). The whiskers indicate the minimum and the maximum values of each experimental group at each time point (1, 3, 6, 24 h). (TIF 844 kb) [file 13041_2016_188_MOESM1_ESM.tif]
